# Synergistic Effect of N Doping and Ag Loading on Photocatalytic Degradation Performance of Rhodamine B by ZnO Nanoarrays

**DOI:** 10.3390/nano16070438

**Published:** 2026-04-02

**Authors:** Congwen Liu, Wei Deng, Hai Zhang, Xiaochen Han, Qiang Ran, Wenxuan Yu, Xiaoling Xu, Zuowan Zhou

**Affiliations:** 1 Key Laboratory of Advanced Technologies of Materials (Ministry of Education), School of Chemistry, Southwest Jiaotong University, Chengdu 610031, China; 18728562632@163.com (C.L.); weideng@my.swjtu.edu.cn (W.D.); zhanghai@swjtu.edu.cn (H.Z.); hanxiaochen1215@163.com (X.H.); 2024201927@my.swjtu.edu.cn (Q.R.); 13994255096@163.com (W.Y.); zwzhou@swjtu.edu.cn (Z.Z.); 2 Yibin Research Institute, Southwest Jiaotong University, Yibin 644000, China

**Keywords:** Ag@N-ZnO nanoarrays, nitrogen doping, silver loading, photocatalytic degradation, rhodamine B

## Abstract

Photocatalytic degradation is a highly efficient, stable and promising technology for water treatment. Developing high-performance photocatalysts is crucial for removing aquatic contaminants. However, traditional zinc oxide (ZnO) photocatalysts are severely restricted by intrinsic drawbacks, such as a wide band gap, fast recombination of photogenerated carriers, and high photocorrosion tendency. Conventional powder catalysts also suffer from difficult recovery and serious secondary pollution. Therefore, developing simple strategies to fabricate high-performance, reusable, and stable ZnO-based photocatalysts is of great scientific and practical importance. In this work, silver-loaded nitrogen-doped ZnO nanoarrays (Ag_Y_@N_X_-ZnO NAs, where X and Y represent the urea and AgNO_3_ concentrations, respectively) were synthesized on 304 stainless steel sheets (SSS) using a two-step hydrothermal method combined with photoreduction at room temperature. The samples were characterized by XRD, FESEM, XPS, and UV-Vis DRS, and the catalytic mechanism was studied through active species trapping and EPR. Nitrogen doping and Ag loading exhibited a strong synergistic effect, narrowing the band gap, enhancing visible-light absorption, and promoting the separation of photogenerated carriers. The optimal sample (Ag_1.5_@N_4_-ZnO NAs) degraded 93.2% of Rhodamine B (RhB) within 180 min, with a reaction rate constant 2.65 times higher than pure ZnO. The main active species were ·O_2_^−^ and ·OH. This work provides a feasible route to fabricate recyclable and stable stainless steel-based ZnO nanoarray photocatalysts for efficient water purification.

## 1. Introduction

With the acceleration of global industrialization and urbanization, refractory organic dye wastewater discharged from printing, dyeing, chemical engineering, and other industries has become a major global water pollution issue, seriously threatening ecological security and human health [1,2,3]. As a typical water-soluble dye, RhB has a stable chemical structure, poor biodegradability and pronounced toxicity. This compound is widely detected in natural water bodies such as rivers and lakes, as well as in industrial discharges [4,5]. Furthermore, it possesses potential carcinogenicity, teratogenicity and mutagenicity, which can cause irreversible damage to the liver, kidney and nervous system of humans and aquatic organisms even at low concentrations, further aggravating its environmental hazard [6]. Therefore, the development of efficient and suitable wastewater treatment technologies is extremely urgent [7,8]. Traditional treatment methods for dye wastewater mainly include physical adsorption [9,10], traditional chemical oxidation methods [11,12,13], and microbial degradation [14,15,16]. Although these methods can remove some dyes, they generally entail high treatment costs, low removal efficiency, and a high risk of secondary pollution [17,18]. Therefore, developing organic wastewater treatment technologies that are green, highly efficient, and capable of complete mineralization is an urgent need in environmental remediation.

Photocatalytic technology, as a typical representative of advanced oxidation processes (AOPs) and driven by solar energy, can fully mineralize refractory organic pollutants into harmless small molecules at room temperature and atmospheric pressure [19,20]. It possesses the merits of no secondary pollution, simple operation, and high sustainability, thereby rendering it one of the most promising strategies for water treatment [21,22]. Zinc oxide (ZnO), a typical n-type wide band gap semiconductor [23], has become a research focus in photocatalysis due to its high electron mobility [24], environmental friendliness, and readily available raw materials [25,26,27]. However, pure ZnO has a wide band gap (~3.37 eV), which can only respond to ultraviolet (UV) light, accounting for less than 5% of solar energy, leading to very low solar efficiency [28]. Additionally, rapid photogenerated carrier recombination, and low quantum efficiency severely limit the intrinsic photocatalytic performance of ZnO. The easy agglomeration of ZnO nanopowders results in difficult post-reaction recovery and potential nanoscale secondary pollution. These inherent drawbacks greatly hinder the large-scale practical application of ZnO [29,30]. To overcome these issues, researchers have enhanced the photocatalytic performance of ZnO through various strategies, including elemental doping [31,32,33], noble metal deposition [34,35,36] and morphology control [37,38]. Specifically, elemental doping can narrow the band gap of ZnO. This modification can further boost the solar utilization efficiency of the material [39]. Noble-metal deposition is another effective strategy to enhance photocatalytic activity. It achieves this effect through the surface plasmon resonance (SPR) effect of noble metal nanoparticles (Au, Ag, Pt, etc.) [36]. Meanwhile, deposited metal particles can form a Schottky barrier at the metal–semiconductor interface. This barrier can effectively increase the separation efficiency of photogenerated electron–hole pairs [40,41].

Compared with traditional powder photocatalysts, semiconductor nanoarray structures offer significant advantages for photocatalytic applications. Vertically aligned and ordered nanoarrays can provide direct, rapid charge–transport pathways, effectively suppressing the recombination of photogenerated carriers and improving charge-separation efficiency [42,43]. Meanwhile, the open, interconnected void structure of nanoarrays enhances mass transfer and exposes more active sites, thus promoting surface catalytic reactions [44,45,46]. More importantly, the integrated nanoarrays grown in situ on the substrate, effectively prevent particle agglomeration and facilitate catalyst recovery [47,48], greatly reducing the risk of secondary pollution and improving cyclic stability [49,50]. Therefore, the construction of ZnO-based nanoarray photocatalysts is an effective strategy to break through the limitations of powder catalysts.

In this work, N-ZnO NAs were grown on a SSS via a hydrothermal method, and Ag nanoparticles were deposited on the N-ZnO NAs via a photoreduction process to enhance photocatalytic performance for RhB degradation. The effects of N-doping concentration and Ag loading on the structure, photoelectrochemical properties, and photocatalytic RhB degradation were systematically studied. Combined with multiple characterization techniques and mechanism analysis, the synergistic enhancement mechanism of N-doping and Ag loading was proposed. This study offers a simple and practical design strategy for developing high-performance, recyclable, and integrated photocatalysts supported on stainless steel, providing theoretical and experimental support for the application of photocatalytic technology in organic wastewater treatment.

## 2. Materials and Methods

### 2.1. Materials

The substrate used in this work was 304 SSS (2 cm × 2 cm) produced by Ruipu Metal Materials Factory (Dongguan, China). Anhydrous ethanol, methanol, and silver nitrate (AgNO_3_) were purchased from Kelong Chemical Co., Ltd. (Chengdu, China); zinc nitrate hexahydrate (Zn(NO_3_)_2_·6H_2_O), hexamethylenetetramine (HMTA, (CH_2_)_6_N_4_) and isopropanol (IPA) were obtained from Aladdin Reagent Co., Ltd. (Shanghai, China). Ethylenediaminetetraacetic acid disodium salt (EDTA-2Na) and 1,4-benzoquinone (BQ) were purchased from Macklin Biochemical Co., Ltd. (Shanghai, China). RhB and zinc acetate (Zn(CH_3_COO)_2_·2H_2_O) were purchased from Sinopharm Chemical Reagent Co., Ltd. (Shanghai, China). All chemical reagents used in this work were of analytical grade and did not require further purification. Deionized water was prepared using a laboratory-specific pure water system for experimental use.

### 2.2. Two-Step Preparation of N_X_-ZnO NAs

N_X_-ZnO NAs were prepared using a two-step process, consisting of two stages: seed-layer formation and hydrothermal growth [51,52]. The steps were as follows:

Preparation of ZnO seed layer: 1.65 g of zinc acetate was weighed and placed in a beaker, with 150 mL of anhydrous ethanol serving as the solvent. It was magnetically stirred in a 60 °C water bath until completely dissolved, forming a homogeneous seed solution at 5 mM. The dried 304 SSS were immersed in the seed solution for 1 min after ultrasonic cleaning with ethanol and deionized water alternately 3 times (3 min each), then removed and dried in a 200 °C electric drying oven for 15 min. The dip-coating and drying cycle described above was repeated four times to produce stainless steel substrate samples with a uniformly loaded seed layer on the surface.

Hydrothermal growth of N_X_-ZnO NAs: Equal molar amounts of zinc nitrate hexahydrate and hexamethylenetetramine, each at a concentration of 50 mM, were weighed and added to deionized water together, followed by magnetic stirring at room temperature (25 °C) for 18–24 h until completely dissolved. Then, a specific amount of urea was weighed and added to the mixture, which was stirred magnetically at room temperature for 1 h to form a homogeneous N_X_-ZnO growth solution (X represents the concentration of urea in the growth solution, with the unit of mM). The solution was divided into 80 mL blue-cap bottles, and the samples with seed layers were suspended vertically in the center of each bottle using a high-temperature-resistant string to ensure full immersion in the growth solution. The blue-cap bottles were placed in a 95 °C electric drying oven for hydrothermal growth for 4 h. After the reaction, the samples were removed, rinsed repeatedly with deionized water and anhydrous ethanol to remove physically adsorbed impurities, and then dried. Finally, the samples were placed in a muffle furnace and annealed at 400 °C for 2 h to eliminate residual organic matter on and within the samples, yielding N_X_-ZnO NAs. The optimal urea concentration in this experiment was 4 mM, and the corresponding concentration exploration process is detailed in the Supplementary Materials.

### 2.3. Preparation of Ag_Y_@N_4_-ZnO NAs by Photoreduction Method

**Figure 1:**
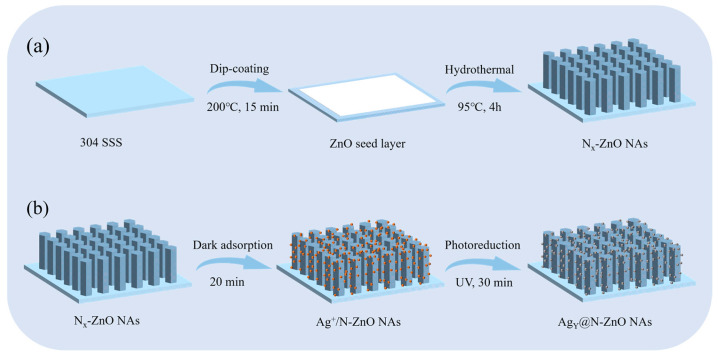
Schematic diagram of the preparation process of Ag_Y_@N_X_-ZnO nanoarrays: (**a**) Preparation of N_X_-ZnO nanoarrays; (**b**) Preparation of Ag_Y_@N_X_-ZnO nanoarrays.

Using N_4_-ZnO NAs prepared under optimal conditions as the substrate, Ag nanoparticles (Ag NPs) were loaded by the photoreduction method to prepare Ag_Y_@N_4_-ZnO NAs composite photocatalytic materials. The specific steps are as follows: First, the N_4_-ZnO NAs samples were rinsed with deionized water and dried to remove surface-adsorbed impurities; different masses of silver nitrate powder were weighed and added to deionized water, forming silver nitrate solutions with concentrations of 0.5 mM, 1.5 mM and 2.5 mM, and methanol was added to the solutions at a volume ratio of V (deionized water):V (methanol) = 20:1. Methanol, as a hole scavenger, can effectively avoid the formation of Ag_2_O by-products during the reduction process. The treated N_4_-ZnO NAs were tiled in a Petri dish, and 1 mL of the above AgNO_3_-mixed solution was uniformly dropped onto the sample surface with a pipette, followed by standing in the dark for 20 min to allow sufficient adsorption of Ag^+^ on the surface of the N_4_-ZnO NAs. Then, a 365 nm LED ultraviolet lamp was turned on, and the light was vertically irradiated onto the sample surface for 30 min to photoreduce Ag^+^ to Ag NPs on the array surface. After the photoreduction reaction, the sample surface was repeatedly rinsed with deionized water to remove unreduced Ag^+^, and finally, the sample was placed in a vacuum drying oven and dried at 60 °C for 2 h to obtain Ag_Y_@N_4_-ZnO composite nanoarray materials. Among them, Y is the concentration of the dropped AgNO_3_-mixed solution. The preparation process is shown in Figure 1.

### 2.4. Characterization

Various characterization methods were used to analyze the prepared samples and to explore their crystal structure, microscopic morphology, surface chemical composition, and valence-state characteristics. X-ray diffractometer (XRD, Bruker D8, Bremen, Germany) was employed for crystal structure analysis, using a copper target as the radiation source (λ = 1.5418 Å), with a scanning angle range of 20–80° and a scanning rate of 20°/min. A field-emission scanning electron microscope (FESEM, ZEISS Sigma 360, Oberkochen, Germany) was used to characterize the nanoarray morphology. An X-ray photoelectron spectrometer (XPS, Thermo Scientific K-Alpha, Oxford, UK) was used to analyze the surface chemical composition and the chemical valence states of the elements in the samples. A UV-Vis spectrophotometer (UV-Vis DRS, Shimadzu UV-3600i Plus, Tokyo, Japan) was used to obtain diffuse reflectance absorption spectra in the wavelength range of 250–600 nm. A fluorescence spectrometer (PL, Edinburgh FLS1000, Livingston, UK) was used to characterize the photoluminescence spectrum, with an excitation wavelength of 325 nm. A UV-Vis spectrophotometer (Shimadzu UV-2600, Tokyo, Japan) was used to measure the absorbance of RhB in the degradation system. An electron spin resonance spectrometer (ESR, JEOL, JES-FA200, Tokyo, Japan) was used to analyze reactive oxygen species (ROS) in the photocatalytic system, using 5,5-dimethyl-1-pyrroline N-oxide (DMPO) as the spin trap for hydroxyl radicals (·OH) and superoxide anions (·O_2_^−^), and 2,2,6,6-tetramethylpiperidine-1-oxyl (TEMPO) as the trapping agent for holes (h^+^).

### 2.5. Electrochemical Testing

A variety of photoelectrochemical characterization methods were used to analyze the prepared samples and explore their photoelectrochemical properties and photogenerated charge-separation characteristics. Electrochemical workstation (CHI660D, Shanghai, China) was used for electrochemical performance evaluation of the prepared samples at room temperature, with a standard three-electrode system where the prepared sample served as the working electrode, a Pt electrode and a Ag/AgCl electrode (saturated KCl) served as the counter electrode and reference electrode respectively, 0.5 M Na_2_SO_4_ aqueous solution as the electrolyte, and a 300 W xenon lamp (Perfectlight, Microsolar 300, Beijing, China) as the irradiation light source; transient photocurrent response and electrochemical impedance spectroscopy (EIS) of the samples were tested to characterize the charge separation and transfer properties of the photocatalysts.

### 2.6. Photocatalytic Degradation Experiments

Using a 5 W LED lamp (wavelength range: 350–800 nm) equipped in a multi-channel photocatalytic reaction system (Perfectlight, PCX50C, Beijing, China) as the light source, the photocatalytic degradation performance of SSS, ZnO NAs, N_4_-ZnO NAs, and Ag_Y_@N_4_-ZnO NAs on RhB was explored. The experimental steps are as follows: 50 mL of a 5 mg/L RhB aqueous solution was prepared, and the sample to be tested was horizontally fixed in the solution to ensure that the light source vertically irradiated the sample surface; magnetic stirring was continued in the dark for 60 min to achieve adsorption–desorption equilibrium of RhB molecules on the sample surface. The light source was then turned on for the degradation reaction, and 3 mL of reaction solution was collected every 30 min. The absorbance of RhB at the characteristic wavelength of 553 nm at different times was measured by a UV-Vis spectrophotometer. To ensure the experiment’s reliability, 3 parallel experiments were run for each sample.

### 2.7. ROS Trapping Experiments

The procedure for the ROS quenching experiment was essentially the same as that for the photocatalytic degradation experiment. RhB was used as the target pollutant for degradation, and EDTA-2Na, BQ and IPA were used as specific scavengers for h^+^, ·O_2_^−^ and ·OH, respectively. The contribution of each active species to the degradation process was determined by comparing the reduction in photocatalytic degradation efficiency upon addition of different scavengers.

## 3. Results and Discussion

### 3.1. Characteristics of the Materials

A series of studies on ZnO NAs has been carried out in previous work [51,52,53], to enhance the photocatalytic degradation performance, nitrogen doping and silver loading were employed to modify ZnO NAs synthesized on 304 stainless steel substrates in this work. First, the effect of nitrogen doping concentration on the morphological structure, energy band structure, and photocatalytic performance of ZnO nanoarrays was systematically investigated. As shown in Figure S1, the average diameter of undoped ZnO nanoarrays was determined to be approximately 95.8 nm, which was gradually reduced to 71.2 nm with the increase in urea concentration. This trend was attributed to the dual role of urea in the reaction system as both the nitrogen source for in situ N doping and the alkali source for regulating the morphology of ZnO nanoarrays, achieving the controllable modulation of the array microstructure. It was shown by XRD characterization results (Figure S2) that the diffraction peak of the (002) crystal plane of ZnO shifted to a lower angle after nitrogen doping, which preliminarily confirmed that lattice distortion of ZnO was induced by nitrogen doping. The results of the N 1s spectrum from XPS characterization (Figure S3) revealed that nitrogen species existed in the ZnO lattice in the form of lattice nitrogen, by which successful incorporation of nitrogen into the ZnO lattice was further verified. The results of UV-Vis diffuse reflectance spectroscopy (UV-Vis DRS, Figure S4) indicated that when the urea concentration was set at 4 mM, an obvious red shift of the optical absorption edge of the sample was observed, corresponding to the effectively narrowed band gap of ZnO.

**Figure 2:**
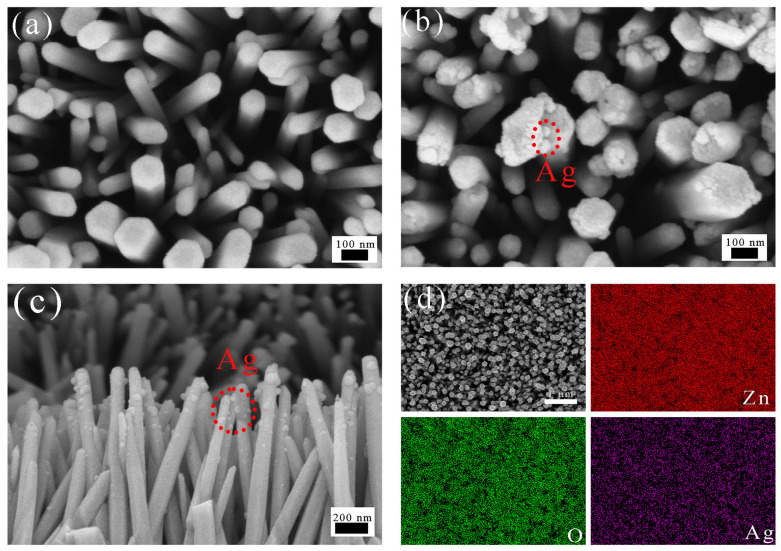
The SEM of photocatalysts: (**a**) SEM images of N-ZnO NAs (**b**) Ag@N-ZnO Nas and (**c**) side view of Ag@N-ZnO NAs, (**d**) EDS images of Ag@N-ZnO NAs.

After thoroughly characterizing the structural and optical properties of samples with different N doping levels, the N-ZnO NAs prepared with a urea concentration of 4 mM were chosen as the substrate for subsequent Ag loading. As shown in Figure 2a, a smooth surface without obvious impurities can be observed for the resulting N-ZnO NAs. Following Ag nanoparticle loading via a room-temperature photoreduction method, numerous uniformly dispersed fine particles were observed on the surface of the nanoarrays (Figure 2b,c). In addition, the elemental composition and spatial distribution of the samples were analyzed using EDS, as shown in Figure 2d. Ag was found to be evenly distributed across the surface of the nanoarrays, along with the intrinsic elements Zn and O. No characteristic N signal was detected in the EDS spectrum, mainly due to the low N doping level and the detection limitations of EDS for light elements.

**Figure 3:**
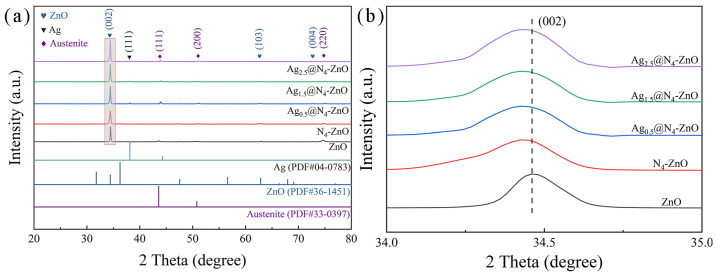
The XRD of photocatalysts: (**a**) Full XRD pattern and (**b**) diffraction peaks of the (002) crystal plane of ZnO NAs, N_4_-ZnO NAs and Ag_Y_@N_4_-ZnO NAs.

Subsequently, the phase structure and crystal orientation of the as-prepared nanoarrays were analyzed by XRD (Figure 3a). For the ZnO NAs without N doping and Ag loading, all diffraction peaks, except those from the austenitic SSS, matched the standard powder diffraction file of wurtzite ZnO (PDF#36-1451) [52]. Notably, the diffraction peak intensity of the (002) crystal plane was much higher than that of the (100) and (101) planes, indicating a preferred growth orientation along the (002) plane, which aligns with the SEM results mentioned earlier. After loading with Ag, new diffraction peaks corresponding to metallic Ag were detected and matched the standard PDF card for metallic Ag (PDF#04-0783) [54], further verifying the successful immobilization of Ag nanoparticles on the surface of N_4_-ZnO NAs.

**Figure 4:**
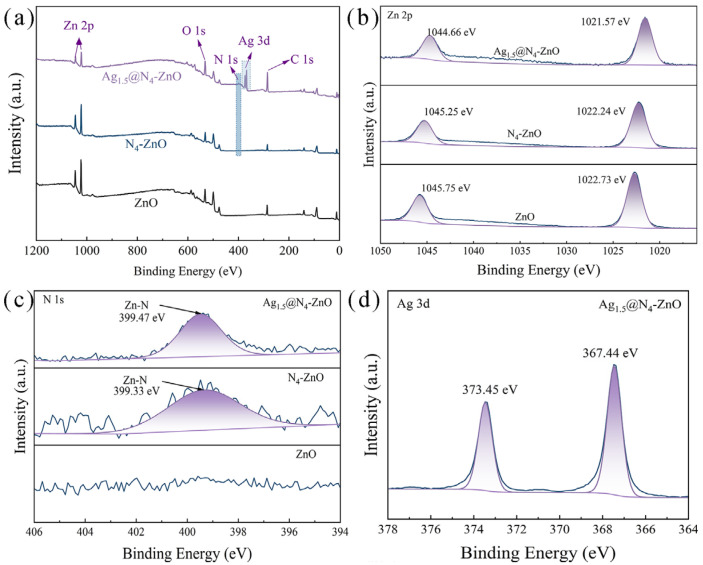
The XPS of photocatalysts: (**a**) XPS survey spectra, (**b**) The Zn 2p and (**c**) N 1s core level XPS spectra of ZnO NAs, N_4_-ZnO NAs and Ag_1.5_@N_4_-ZnO NAs, (**d**) Ag 3d core level XPS spectra of Ag_1.5_@N_4_-ZnO NAs.

Successively, XPS was conducted to systematically analyze the surface elemental composition and chemical states of the as-prepared nanoarrays. As shown in the full survey spectrum (Figure 4a), only characteristic peaks corresponding to Zn and O were observed on the surface of pristine ZnO NAs. An additional N 1s peak was detected in the spectrum of N-ZnO NAs, while distinct Ag 3d peaks were detected after loading Ag nanoparticles, which aligns with the design of the material system. The high-resolution Zn 2p spectra of the as-prepared samples are shown in Figure 4b. For pristine ZnO, two symmetric peaks at binding energies of 1045.75 eV and 1022.73 eV were observed, assigned to the Zn 2p^1/2^ and Zn 2p^3/2^ spin–orbit orbitals, respectively. The spin–orbit splitting energy of 23.02 eV between these peaks confirms that Zn species in the material are in the divalent Zn^2+^ form [55,56]. Notably, the binding energies of the Zn 2p peaks shifted significantly after N doping and Ag loading, attributed to changes in the chemical environment of the Zn species. This phenomenon indicates a strong electronic interaction between N, Ag species, and ZnO NAs, rather than simple physical mixing.

The N 1s spectrum of N-ZnO NAs was shown in Figure 4c. After N doping, two clear N 1s peaks appeared at 399.33 eV and 399.47 eV, indicating lattice nitrogen in the form of Zn-N bonds [57,58,59]. Consistent with the peak shift observed in the XRD patterns into the ZnO lattice, which aligns with the peak shift in the (002) crystal plane seen in the previous XRD patterns. Figure 4d shows the high-resolution Ag 3d spectrum of the Ag-loaded sample. Two characteristic peaks are observed at 373.45 eV and 367.44 eV, corresponding to the Ag 3d^3/2^ and Ag 3d^5/2^ orbitals of metallic Ag^0^, with a spin–orbit splitting energy of 6.01 eV [60,61]. This provides strong evidence that Ag nanoparticles are successfully loaded onto the surface of N-ZnO NAs in their metallic form.

### 3.2. Photoelectric Chemical Performance Analysis

**Figure 5:**
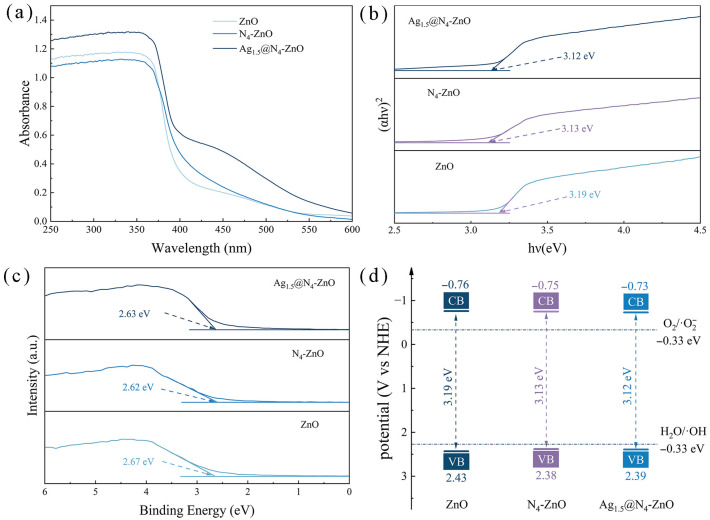
The XPS spectra, UV-Vis spectra and Energy band structure diagrams of photocatalysts: (**a**) UV-Vis diffuse reflectance spectra, (**b**) Tauc plots, (**c**) VB-XPS spectra and (**d**) Energy band structure diagrams of ZnO NAs, N_4_-ZnO NAs and Ag_1.5_@N_4_-ZnO NAs.

To examine how Ag loading influences the light-absorption performance and energy band structure of ZnO nanoarrays, UV-Vis DRS and XPS were used to characterize the samples, as shown in Figure 5. As seen in Figure 5a, a red shift in the UV-Vis absorption edge can be observed for the nanoarrays after Ag NPs loading, and their light absorption capacity in the 400~600 nm range is notably enhanced. This is mainly due to the SPR of Ag NPs, with Ag_1.5_@N_4_-ZnO showing the strongest SPR. The increased light absorption allows the material to capture more light energy during photocatalysis, generate more electron–hole pairs, and thus accelerate the reaction.

The band gaps of ZnO, N_4_-ZnO NAs, and Ag_1.5_@N_4_-ZnO NAs calculated using Equation (1) [62] are 3.19 eV, 3.13 eV, and 3.12 eV, respectively (Figure 5b).

(1) $${\left(\alpha h\nu \right)}^{1/n}=A(h-E_{g})$$

where α denotes the absorption coefficient, *h* is Planck’s constant, *ν* represents the electron frequency, and, e.g., is the band gap of the semiconductor. The value of *n* depends on the intrinsic properties of the semiconductor: *n* is 1/2 for direct band gap semiconductors and 2 for indirect band gap semiconductors. Since ZnO is a common direct band gap semiconductor, *n* is set to 1/2 in this equation. As shown in Figure 5b, band gap narrowing of ZnO is achieved after N doping and Ag loading. This is because non-metallic N doping is incorporated into the ZnO lattice, forming intermediate energy levels within the ZnO band gap, which reduces the energy barrier for electron transition and thus narrows the band gap.

The generation of ROS during photocatalysis is a crucial factor influencing performance and is closely linked to the valence band (VB) and conduction band (CB) potentials of the material. The VB potentials of various catalysts were determined from XPS valence-band spectra. The VB potentials of ZnO, N_4_-ZnO NAs, and Ag_1.5_@N_4_-ZnO NAs are 2.67 eV, 2.62 eV, and 2.63 eV, respectively (Figure 5c) [63]. The VB potentials obtained from XPS were converted to potentials relative to the normal hydrogen electrode (NHE) using Equation (2). The calculated VB potentials of ZnO, N_4_-ZnO NAs, and Ag_1.5_@N_4_-ZnO NAs relative to NHE are 2.43 eV, 2.38 eV, and 2.39 eV, respectively.

(2) $$E_{(\mathrm{VB},\;\mathrm{NHE})\;}={\;E}_{\left(\mathrm{VB},\;\mathrm{XPS}\right)}+\Phi -4.44$$

In Equation (2), *E*_(*VB*, *NHE*)_ is the valence band potential, relative to the normal hydrogen electrode; *E*_(*VB*, *XPS*)_ is the valence band potential obtained from the XPS valence band spectrum; *Φ* is the work function of the XPS instrument, which is 4.20 eV in this experiment.

Combining the band gap and the VB potential relative to NHE, the CB potentials of the materials relative to NHE were calculated by Equation (3). The CB potentials of ZnO, N_4_-ZnO NAs, and Ag_1.5_@N_4_-ZnO NAs are −0.76 eV, −0.75 eV, and −0.73 eV, respectively.

(3) $$E_{g}=E_{\left(\mathrm{VB},\;\mathrm{NHE}\right)}-E_{\left(\mathrm{CB},\;\mathrm{NHE}\right)}$$

where *E*_(*CB*, *NHE*)_ is the conduction band potential relative to the normal hydrogen electrode.

Based on the above experimental data, a schematic diagram of the energy band structure of different samples was created (Figure 5d). The CB potentials of all samples are more negative than the one-electron reduction potential of O_2_/·O_2_^−^ (−0.33 V vs. NHE) [64], which thermodynamically allows the photogenerated electrons on the CB to reduce dissolved O_2_ to ·O_2_^−^. Meanwhile, the VB potentials of all samples are higher than the oxidation potential of ·OH/H_2_O (2.27 V vs. NHE) [54], so the photogenerated holes on the VB can oxidize H_2_O in the solution to ·OH, providing enough reactive oxygen species for photocatalytic degradation.

As shown in Figure S5, the PL intensity of ZnO nanoarrays decreased significantly after Ag loading, with the lowest fluorescence intensity exhibited by Ag_1.5_@N_4_-ZnO NAs. This indicates the most effective suppression of photogenerated carrier recombination and the highest utilization efficiency [65,66]. As depicted in Figure S6a, no photocurrent was produced by any catalyst in the dark, while a stable photocurrent quickly appeared upon light exposure, confirming light-induced electron generation [67]. Among them, the largest photocurrent density and the greatest production of photogenerated electrons were displayed by Ag_1.5_@N_4_-ZnO NAs. As shown in Figure S6b, the arc radius of ZnO nanoarrays decreased after Ag loading, thereby reducing interfacial charge-transfer resistance and accelerating charge transfer [68,69]. In summary, the synergistic effect of Ag loading and N doping reduces carrier recombination in ZnO nanoarrays, thereby increasing the photogenerated electron yield and accelerating charge transfer under light irradiation.

### 3.3. Photocatalytic Degradation Performance

**Figure 6:**
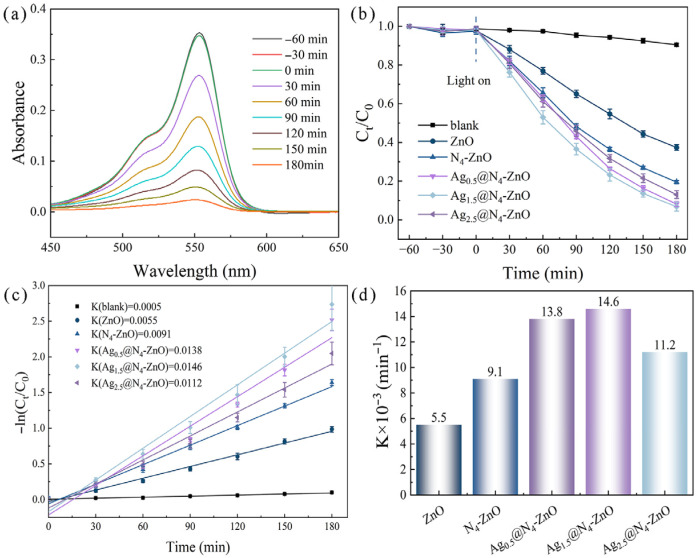
Analysis of photocatalytic degradation performance of Ag_Y_@N_4_-ZnO NAs for RhB: (**a**) UV-Vis absorption spectra of RhB; (**b**) Photocatalytic degradation rate of RhB; (**c**) Pseudo-first-order kinetic curves; (**d**) Reaction rate constants.

The results of photocatalytic degradation experiments at different nitrogen doping concentrations (Figure S7) showed that the as-prepared ZnO nanoarrays achieved the optimal degradation performance toward RhB at a urea concentration of 4 mM. Subsequently, the photocatalytic degradation performance towards RhBof ZnO NAs, N_4_-ZnO NAs and Ag_Y_@N_4_-ZnO Nas, prepared with different concentrations of AgNO_3,_ was compared, and the corresponding experimental results are presented in Figure 6. The UV-Vis absorption spectra of RhB during the photocatalytic reaction are displayed in Figure 6a. As irradiation time increases, the intensity of RhB’s characteristic absorption peak at 553 nm steadily decreases, with no new peaks forming.

The curves of the photocatalytic degradation rate of RhB by different samples as a function of irradiation time are shown in Figure 6b. Within 180 min of irradiation, the degradation rate of RhB by pure ZnO NAs is only 62.3%. After N doping, the degradation rate of N_4_-ZnO NAs is increased to 80.5%, indicating that N doping can effectively improve the photocatalytic performance of ZnO. After loading with Ag, the degradation performance of the samples is further enhanced: the degradation rates of Ag_0.5_@N_4_-ZnO NAs, Ag_1.5_@N_4_-ZnO NAs, and Ag_2.5_@N_4_-ZnO NAs are 91.8%, 93.2%, and 86.9%, respectively. With increasing Ag loading, the photocatalytic degradation performance of the as-prepared samples shows a trend of first increasing and then decreasing, among which Ag_1.5_@N_4_-ZnO NAs exhibit the optimal catalytic activity. When Ag loading exceeds the optimal value, excessive Ag loading induces agglomeration of Ag nanoparticles on the surface of the nanoarrays, which, in turn, masks the active sites on the ZnO surface and ultimately leads to a decline in the material’s photocatalytic degradation performance.

The pseudo-first-order kinetic curves for the photocatalytic degradation of RhB was shown in Figure 6c. A good linear relationship was displayed by all samples’ kinetic curves, indicating that the degradation process follows a pseudo-first-order kinetic model. The corresponding reaction rate constant k, which directly reflects the photocatalytic degradation rate, is presented in Figure 6d. The *k* values for pure ZnO NAs, N_4_-ZnO NAs, Ag_0.5_@N_4_-ZnO NAs, Ag_1.5_@N_4_-ZnO NAs, and Ag_2.5_@N_4_-ZnO NAs are 0.0055, 0.0091, 0.0138, 0.0146 and 0.0112 min^−1^, respectively. The maximum k value of Ag_1.5_@N_4_-ZnO NAs is 2.65 times higher than that of pure ZnO NAs. The trend in the kinetic constant matches the degradation rate, confirming that 1.5 mM AgNO_3_ is the optimal concentration for preparing Ag_Y_@N_4_-ZnO NAs. Excessive Ag acts as a recombination center for photogenerated carriers, decreasing the separation efficiency of electron–hole pairs and reducing the photocatalytic degradation rate. As illustrated in Figure S8, a RhB degradation efficiency over 80% was maintained by the material after 5 cycling runs. XRD and XPS results verified no obvious alterations in its crystal structure, chemical composition and elemental valence states post cycling, confirming the excellent catalytic and structural stability of Ag@N-ZnO NAs. The photocatalytic degradation activity of Ag_1.5_@N_4_-ZnO NAs toward rhodamine exceeds that of most reported photocatalysts (as shown in Table S1), further confirming its effectiveness in degrading rhodamine.

### 3.4. Ag@N-ZnO Photocatalytic Mechanism

**Figure 7:**
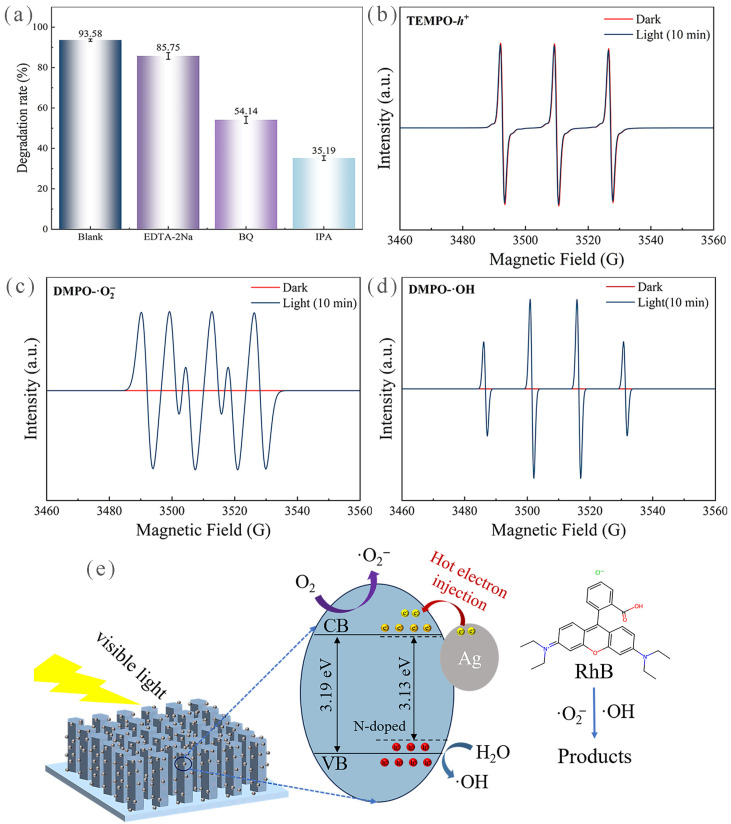
Results of reactive oxygen species detection experiments: (**a**) Reactive oxygen species trapping experiments, (**b**) TEMPO-h^+^EPR spectrum, (**c**) DMPO-·O_2_^−^ EPR spectrum and (**d**) DMPO-·OH EPR spectrum of Ag_1.5_@N_4_-ZnO NAs; (**e**) Schematic diagram of the photocatalytic degradation mechanism of Ag@N-ZnO NAs.

To clarify the mechanism of photocatalytic degradation, the behavior of ROS generation on the prepared samples was systematically examined through multiple experiments. First, the production of ·OH and ·O_2_^−^ under light exposure was qualitatively and visually confirmed, using the TMB and NBT methods, respectively (Figures S9 and S10). Next, ROS trapping experiments were conducted to measure each active species’ contribution during degradation, using EDTA-2Na, BQ and IPA as scavengers for h^+^, ·O_2_^−^, and ·OH, respectively. As shown in Figure 7a, the photocatalytic degradation efficiency decreased to different extents with the addition of various scavengers. Notably, the largest decreases in efficiency were exhibited by the groups with BQ and IPA, indicating that ·O_2_^−^ and ·OH are the primary active species responsible for photocatalytic degradation. Finally, direct qualitative verification of ROS production was achieved through EPR measurements, where characteristic peaks of ·O_2_^−^ and ·OH signals were clearly observed (Figure 7b–d), confirming that the photocatalytic degradation by Ag_1.5_@N_4_-ZnO nanoarrays is mainly driven by ·O_2_^−^ and ·OH.

Based on the comprehensive results from the energy band structure, photoelectrochemical performance, ROS detection, and EPR measurements described above, a synergistic mechanism for the enhanced photocatalytic degradation of RhB by the Ag@N-ZnO composite photocatalyst was proposed (Figure 7e). The core of this mechanism lies in the synergistic modulation effect of N doping and Ag NP loading. Specifically, the intrinsic band gap of ZnO was decreased from 3.19 eV to 3.13 eV by N doping. It not only created impurity intermediate energy levels within the band gap to lower the transition energy barrier for photogenerated electrons, but also induced lattice distortion of the (002) active crystal plane, increasing the number of active sites on the material surface [70,71]. The SPR effect of Ag NPs further reduced the composite’s band gap to 3.12 eV, greatly improving light absorption in the visible range (400–600 nm) and simultaneously boosting the efficient generation of hot electrons. The Schottky barrier at the Ag/N-ZnO interface enabled metallic Ag to serve as an effective trapping site for photogenerated electrons, promoting rapid injection of hot electrons and efficient separation of electron–hole pairs. According to energy band potential calculations, the CB potentials of the prepared materials ranged from −0.73 to −0.76 eV versus the NHE, which are more negative than the reduction potential of ·O_2_^−^/O_2_ (−0.33 eV vs. NHE). Meanwhile, the VB potentials ranged from 2.38 to 2.43 eV vs. NHE, which are more positive than the oxidation potential of ·OH/H_2_O (2.27 eV vs. NHE). This band structure ensures thermodynamically that the photogenerated electrons in the CB can reduce dissolved oxygen to ·O_2_^−^, while the photogenerated holes in the VB can oxidize water molecules to ·OH. Combined with the results from EPR and ROS trapping experiments, these two ROS were identified as the key oxidative active species in the photocatalytic process. Their strong oxidation ability can effectively break down the conjugated chromophore structure of RhB, leading to deep mineralization and degradation of the pollutant.

## 4. Conclusions

In summary, recyclable Ag_1.5_@N_4_-ZnO NAs were successfully synthesized on 304 stainless steel substrates using a simple dip-coating assisted hydrothermal method, followed by room-temperature photoreduction. The optimal conditions were identified as 4 mM urea for nitrogen doping and 1.5 mM AgNO_3_ for silver loading. Compared to pristine ZnO and N_4_-ZnO NAs, the photocatalytic RhB degradation ability of the composite was greatly improved by the synergistic effect of N doping and Ag loading. 93.2% degradation of 5 mg/L RhB was achieved by the optimal Ag_1.5_@N_4_-ZnO NAs within 180 min., with a reaction kinetic constant 2.65 times higher than that of pristine ZnO. Additionally, the composite kept excellent structural and cycling stability after multiple uses. The improved catalytic performance results from band gap narrowing of the arrays induced by N doping, along with significantly enhanced photoelectrochemical performance driven by the SPR effect of Ag NPs. EPR, reactive oxygen species trapping, and active species detection experiments confirmed that ·O_2_^−^ and ·OH are the main reactive species involved in RhB degradation. A simple and practical strategy for fabricating recyclable stainless-steel-supported ZnO-based photocatalysts is presented in this work, and the as-prepared composite demonstrates promising potential for organic wastewater treatment. In future work, the research scope of target pollutants will be expanded, the photocatalytic degradation performance of this as-prepared catalyst toward priority pollutants will be focused on, and its practical application value in the treatment of actual environmental wastewater will be further verified.

## Data Availability

The original contributions presented in this study are included in the article/Supplementary Materials. Further inquiries can be directed to the corresponding author.

## Supplementary Materials

The following supporting information can be downloaded at https://www.mdpi.com/article/10.3390/nano16070438/s1, The Reactive Oxygen Species Detection Experiments; Cycling Stability Experiments; Figure S1: Effect of urea concentration on the morphology of ZnO NAs; Figure S2: Effect of urea concentration on the crystal structure of ZnO NAs; Figure S3: The XPS of photocatalysts: (a) XPS survey spectra, (b) Zn 2p, (c) O 1s, and (c) N 1s core level XPS spectra of ZnO NAs and N_4_-ZnO NAs; Figure S4: Optical properties and energy band structure of the catalyst: (a) UV-Vis diffuse reflectance spectrum; (b) band gap; (c) XPS valence band spectrum; (d) schematic diagram of the energy band structure; Figure S5: PL spectra of Ag_Y_@N_4_-ZnO NAs; Figure S6: Photoelectrochemical properties of ZnO, N_4_-ZnO, Ag_1.5_@N_4_-ZnO (a) Photocurrent density curves; (b) electrochemical impedance spectroscopy; Figure S7: Analysis of photocatalytic degradation performance of N_4_-ZnO NAs for RhB: (a) UV-Vis absorption spectra of RhB; (b) Photocatalytic degradation rate of RhB; (c) Pseudo-first-order kinetic curves; (d) Reaction rate constants; Figure S8: Cycling stability tests of Ag_1.5_@N_4_-ZnO NAs: (a) Cycling performance; (b) XRD patterns; (c) XPS spectra; Figure S9: Detection of ·OH generation from Ag_1.5_@N_4_-ZnO NAs by TMB method: (a) UV-visible absorption spectra of TMB; (b) Absorbance changes in TMB; (c) Digital images of sample solutions before and after reaction; Figure S10: Detection of ·O_2_^−^ generation by NBT method: (a) Effect of light irradiation on NBT; (b) UV-visible absorption spectra of NBT (Ag_1.5_@N_4_-ZnO NAs); (c) Degradation curves of NBT; (d) Digital images of samples before and after reaction; Table S1: The comparison of some recent literature reports on the photocatalytic degradation of RhB by various photocatalysts with our study. References [72,73,74,75,76,77,78,79] are cited in the Supplementary Materials.
