# Supplementary material for: Synergistic Effect of N Doping and Ag Loading on Photocatalytic Degradation Performance of Rhodamine B by ZnO Nanoarrays
Source: Nanomaterials (Basel). 2026 Apr 2;16(7):438. doi: 10.3390/nano16070438 (PMC13074961; doi:10.3390/nano16070438)
Supplement: Supplementary file 1 [file nanomaterials-16-00438-s001.zip › nanomaterials-4232026-supplementary.pdf]

Synergistic Effect of N-Doping and Ag-Loading on Photocatalytic Degradation Performance of Rhodamine B by ZnO Nanoarrays

Congwen Liu[1], Wei Deng[1], Hai Zhang[1], Xiaochen Han[1], Qiang Ran[1], Wenxuan Yu[1], Xiaoling Xu[1,2,]*and Zuowan Zhou[1,2]

[1]Key Laboratory of Advanced Technologies of Materials (Ministry of Education), School of Chemistry, Southwest Jiaotong University, Chengdu, 610031, China

[2]Yibin Research institute, Southwest Jiaotong University, Yibin 644000, China

*Correspondence: bihan_2001@163.com; Tel.: +86-13880418330

1. Methods

1.1 Reactive Oxygen Species Detection Experiments

(1) NBT Assay for Superoxide Radical ($\cdot O_2^-$)

Nitro blue tetrazolium chloride (NBT) was first dissolved to 2.5 mmol/L in aqueous solution, which was used to evaluate the photocatalyst's generation of superoxide anions ($\cdot O_2^-$). The NBT aqueous solution has a characteristic maximum absorption peak at 259 nm. NBT can be reduced by $\cdot O_2^-$, leading to a decrease in its absorbance at this wavelength. The faster the absorbance at 259 nm decreases within the same irradiation duration, the more $\cdot O_2^-$ is generated in the photocatalytic system. One piece of the as-prepared photocatalyst was added to 50 mL of the above NBT aqueous solution, and the mixture was stirred under a xenon lamp. Water samples were collected every 10 min, and the absorption spectra were scanned with a UV-Vis spectrophotometer to plot the absorbance versus irradiation time.

(2) TMB Assay for Hydroxyl Radical ($\cdot OH$)

3,3',5,5'-Tetramethylbenzidine (TMB) was first prepared into a 2.5 mmol/L aqueous solution. The as-prepared photocatalyst sample was fixed in the above solution and irradiated under simulated sunlight. The UV-Vis absorption spectrum of the TMB solution was tested every 10 min, and the absorbance at 652 nm was recorded to detect the generation of hydroxyl radicals ($\cdot OH$) in the photocatalytic system.

1.2 Cycling Stability Experiments

The cycling stability test was performed by repeating the photocatalytic degradation experiment for multiple runs. The degradation efficiency of each cycle was calculated to evaluate the attenuation

of catalytic performance. After cycling, the used catalyst samples were characterized by XRD and XPS to analyze the changes in their crystal structure and surface chemical composition.

2. Results

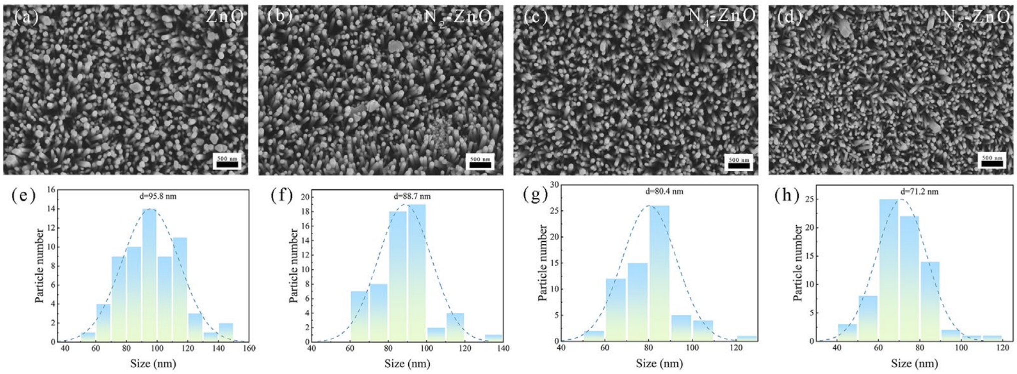

Figure S1. Effect of urea concentration on the morphology of ZnO NAs.

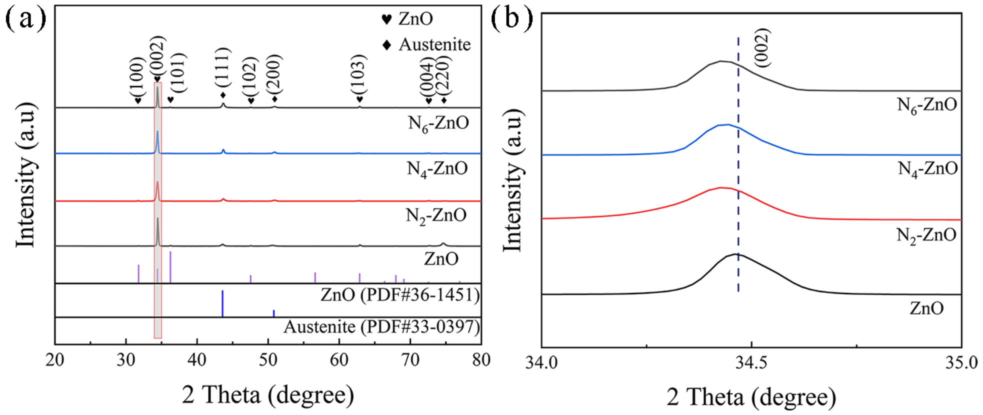

Figure S2. Effect of urea concentration on the crystal structure of ZnO NAs.

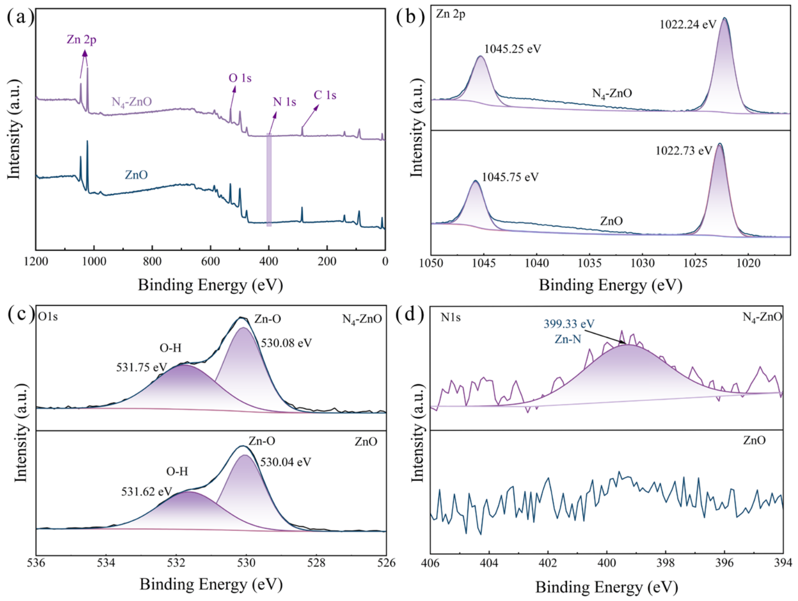

Figure S3 The XPS of photocatalysts: (a) XPS survey spectra, (b) Zn 2p, (c) O 1s, and (c) N 1s core level XPS spectra of ZnO NAs and N_4-ZnO NAs.

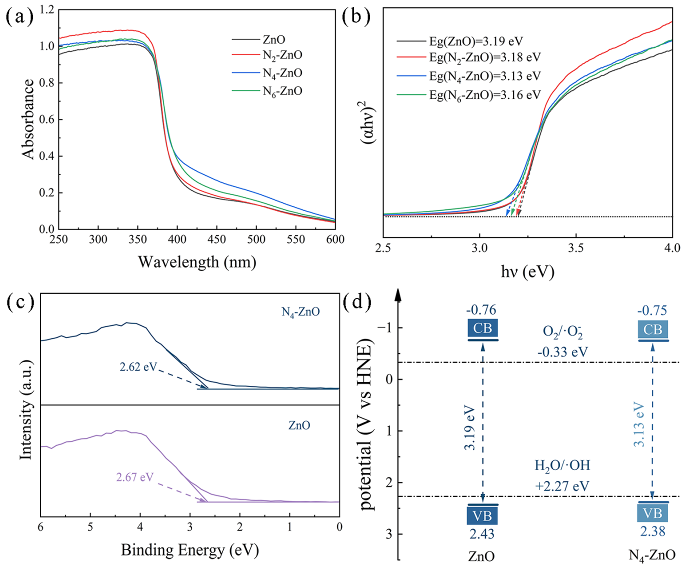

Figure S4. Optical properties and energy band structure of the catalyst: (a) UV-Vis diffuse reflectance spectrum; (b) band gap; (c) XPS valence band spectrum; (d) schematic diagram of the energy band structure.

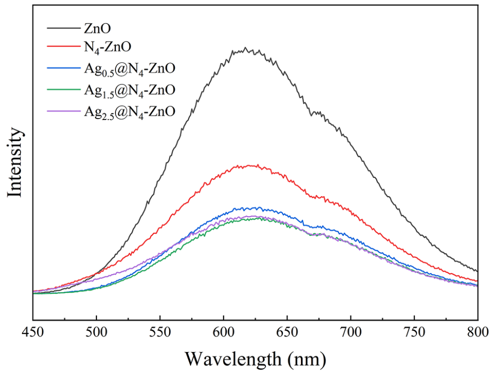

Figure S5. PL spectra of Ag_Y@N_4-ZnO NAs

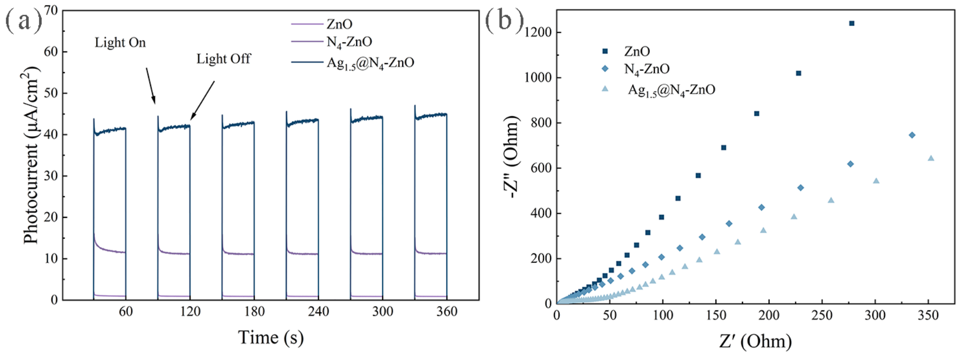

Figure S6. Photoelectrochemical properties of ZnO, N_4-ZnO, $Ag_{1.5}$@N_4-ZnO: (a) Photocurrent density curves; (b) electrochemical impedance spectroscopy

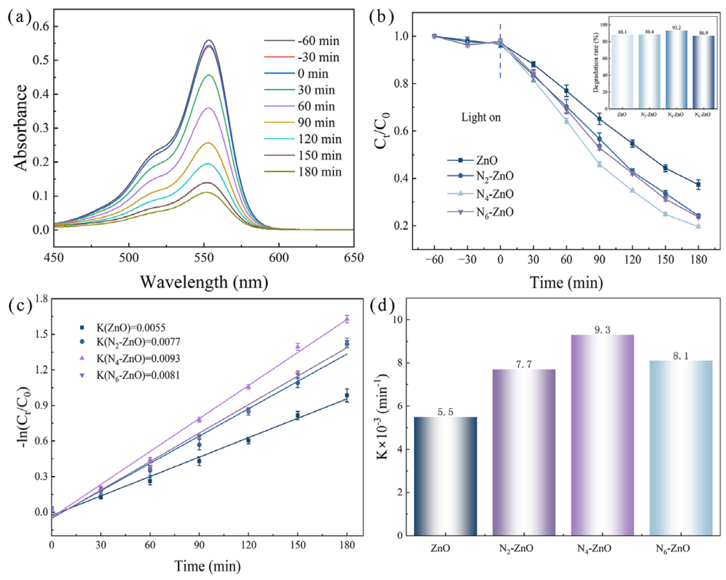

Figure S7. Analysis of photocatalytic degradation performance of N_4-ZnO NAs for RhB: (a) UV-Vis absorption spectra of RhB; (b) Photocatalytic degradation rate of RhB; (c) Pseudo-first-order kinetic curves; (d) Reaction rate constants.

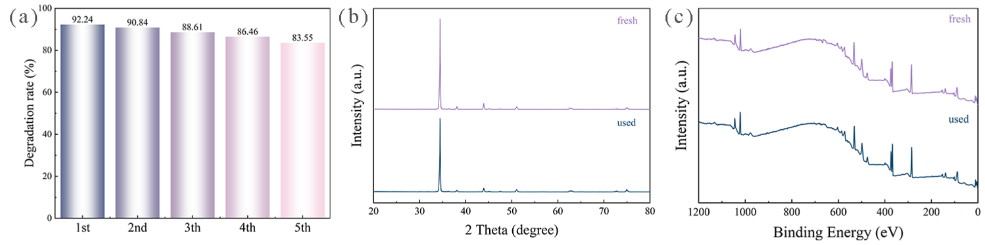

Figure S8. Cycling stability tests of $Ag_{1.5}$@N_4-ZnO NAs: (a)Cycling performance; (b) XRD patterns; (c) XPS spectra.

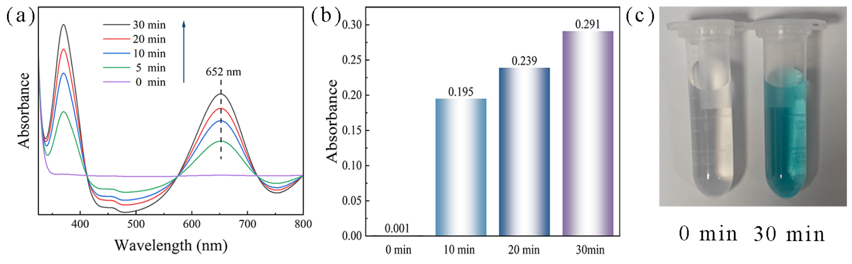

Figure S9. Detection of ·OH generation from $Ag_{1.5}$@N_4-ZnO NAs by TMB method: (a) UV-visible absorption spectra of TMB; (b) Absorbance changes of TMB; (c) Digital images of sample solutions before and after reaction.

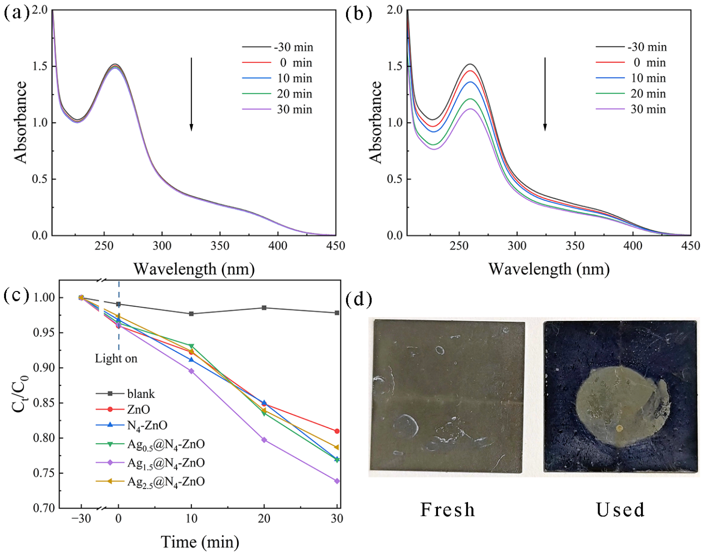

Figure S10. Detection of $\cdot O_2^-$ generation by NBT method: (a) Effect of light irradiation on NBT; (b) UV-visible absorption spectra of NBT ($Ag_{1.5}$@N_4-ZnO NAs); (c) Degradation curves of NBT; (d) Digital images of samples before and after reaction

Table S1. The comparison of some recent literature reports on the photocatalytic degradation of RhB by various photocatalysts with our study

Photocatalysis	Light source	The concentration of RhB (mg/L)	The concentration of Catalysis (g/L)	Degradation efficiency	Ref.
Ag@N-ZnO NAs	5W LED lamp 350-800 nm	5	0.1	93.2% in 180 min	**This work**
ZnO Nanoplates	250 W visible-light lamp	20	0.4	28.0% in 60min	[1]
C-doped ZnO	400 W halogen lamp	4	0.5	80.0% in 300 min	[2]
ZnO NPs	visible light	20	0.1	67.0% in 150 min	[3]
Fe_3O_4/ZnO@MXene	300W Xenon-lamp λ> 400 nm	10	1.0	98.4% in 120 min	[4]
r-TiO_2@CCB	xenon lamp	5	-	90.0% in 180 min	[5]

$MoS_2/CaTiO_3$	15W LED lamp (6500K)	1	0.1	97.0% in 180 min	[6]
ZnO@GAC	UV-A	5	0.1	82.0% in 120 min	[7]
Zn_2SnO_4/SnO_2	-	10	1.0	70.6% in 120 min	[8]

References

1. Meena, P.L.; Meena, R.; Meena, J.; Meena, S.; Surela, A.K.; Kumar, N.; Dhanetia, H.R.; Selvaraj, M. Green synthesis of spherical ZnO nanoplates possessing lower band gap energy and higher photocatalytic performance. *Mater. Res. Bull.* **2026**, *198*, 113996.
2. Stefan, M.; Toloman, D.; Ammar, A.U.; Rostas, A.M.; Macavei, S.; Bocirnea, A.E.; Vasile, B.S.; Perhaita, I.; Popa, A. Defective C-doped ZnO with enhanced photocatalytic and supercapacitor performances. *Ceram. Int.* **2026**, *52*, 960–973.
3. Phan, T.L.; Tu, B.D.; Ho, T.A.; Quang, T.V.; Petrov, D N.; Huy, B.T.; Kim, D.H.; Dang, N.T. Enhanced magnetic ordering, and microwave-shielding and photocatalytic performance in hydrogenated ZnO nanoparticles. *Appl. Surf. Sci.* **2025**, *690*, 162636.
4. An, X.; Ding, H.; Wang, Y.;Fan, B.B.; Li, M.L.; Shao, G.; Xu, H.L.; Wang, H.L.; Lu, H.X. Microwave-assisted one-step synthesis of Fe3O4/ZnO@MXene multi-dimensional composite for photocatalysis and electromagnetic wave absorption. *Appl. Surf. Sci.* **2025**, *706*, 163532.
5. Zhang, C.; Jia, Y.; Chen, X.; Liu, Y.H.; Zhang, L.D.; Zhang, Y.H.From agricultural waste to device: Corncob-derived biocarbon for coupled solar evaporation and photocatalysis. *J. Environ. Manag.* **2026**, *401*, 129013.
6. Luo, M.; Xu, J.; Xu, W.; Zheng, Y.; Wu, G.D.; Jeong, T. Photocatalytic activity of MoS2 nanoflower-modified CaTiO3 composites for degradation of RhB under visible light. *Nanomaterials* **2023**, *13*, 636.
7. Obayomi, K.S.; Lau, S.Y.; Xie, Z.; Gray, S.R.; Zhang, J.H.In-situ hydrothermal fabrication of ZnO-loaded GAC nanocomposite for efficient Rhodamine B dye removal via synergistic photocatalytic and adsorptive performance. *Nanomaterials* **2024**, *14*, 1234.
8. Nikolic, M.V.; Vasiljevic, Z.Z.; Dimitrijevic, M.; Radmilovic, M.; Vujancevic, J.; Tanovic, M.; Tadic, N.B. Natural sunlight driven photocatalytic degradation of methylene blue and rhodamine b over nanocrystalline Zn_2SnO_4/SnO_2. *Nanomaterials* **2025**, *15*, 1138.
